# Supplementary material for: A Qualitative Study on the Impact of First Steps—A Peer-led Educational Intervention for People Newly Diagnosed with Parkinson’s Disease
Source: Behav Sci (Basel). 2019 Oct 10;9(10):107. doi: 10.3390/bs9100107 (PMC6826464; doi:10.3390/bs9100107)
Supplement: Supplementary file 1 [file behavsci-09-00107-s001.pdf]

Supplementary file

This supplementary File contains the following:

Appendix A: The semi-structured interview.
Appendix B: The demographics of study participants
Appendix C: The final stage synthesis

Process Evaluation Interview Schedule
Version 2.0

ID:____

Date: …/…/……..

Closed Questions:
How do you feel now compared to the start of First Steps?
Better / worse / same

Was it worth taking part in First Steps?
Yes/no
Open Questions:
Please could you identify your reflections of the following:

- What did you think of the venue?
- What did you think of the competence and manner of staff?
- What did you think of the scheduling of the session?
    - How did it fit in with your lifestyle?
    - Did you have to make any changes to your lifestyle?
    - Would there be a better time for the session?
- What did you think of the content of the sessions?
- What did you think of the content around exercise?
    - Do you have experiences or stories of physical activity before the programme? Have these stories changed now you have finished the programme?
    - Was physical activity a part of your daily living before the programme? Can you describe any changes following the programme?
    - Do you have any limits of physical activity? Has participation changed this?
    - Do you tell anyone else what you are doing in relationship to physical activity?
- What did you think about the content of the programme around medical management?
    - Did the program have any impact on your medical management?
- What did you think about the content of the programme around getting on with your life?
    - Did the content have any impact?
    - What has doing the programme meant for your life?
    - Was there value in meeting other people with Parkinson's?
- Did the content of the programme impact your motivation:
    - In terms of your motivation and physical activity?
    - In terms of other activities?
- The driving content and impact
    - Did the program aid your driving? For example some people can experience symptoms which influence driving like fatigue.
- Was there any perceived benefits?

    - If so what were they?
- Was there any perceived negative effects or aspects of the programme?
    - If so what were they?

- Did the content of the programme impact the way you manage your Parkinson’s?

- Did the content of the programme impact your confidence?

- Did the content of the programme impact your mood/anxiety?

- Did the content of the programme impact your family or work/voluntary activities?

- Is there anything else you would like to address with regards to your participation in this study?

Table 1 The demographics of included participants

ID	Gender of participant	intervention	Age Years	Time since diagnosis at enrolment (months)
P1F61	F	Y	61	7.00
P2M68	M	Y	68	12.00
P3F77	F	N	77	4.00
P4M83	M	N	83	5.00
P5M75	M	Y	75	5.00
P6M64	M	Y	64	6.00
P7F63	F	Y	63	3.00
P8F70	F	Y	70	9.00
P10M63	M	N	63	9.00
P9M74	M	N	74	3.00
P11M62	M	N	62	7.00
P13M84	M	N	84	1.00
P14M69	M	Y	69	3.00
P15F61	F	N	61	1.00
P18F57	F	Y	57	8.00
P16M69	M	Y	69	1.00
P17F70	F	Y	70	9.00
P19M55	M	Y	55	5.00
P20M72	M	N	72	3.00
P21M73	M	N	73	1.00
P23F54	F	N	54	7.00
P24M76	M	Y	76	3.00
P22F49	F	N	49	10.00

P26M83	M	Y	83	1.00
P35F59	F	Y	59	11.00
P32M60	M	Y	60	1.00
P36F58	F	Y	58	7.00
P38M63	M	Y	63	9.00
P34F74	F	N	74	4.00
P48M80	M	N	80	3.00
P37M71	M	Y	71	4.00
P50F60	F	N	60	11.00
P47M60	M	N	60	3.00
P43M60	M	N	60	6.00
P51F60	F	N	60	9.00
P54F56	F	N	56	10.00
P59F67	F	N	67	8.00
P64M71	M	N	71	8.00
P74M70	M	N	70	5.00
P75M68	M	N	68	5.00

Synthesis of qualitative data

Narrative Responses to the group

Theme	Definition	Example Unit
**Impact on the illness narratives**	The affirmed narrative; no real impact Knew already what to do or nothing I didn't already know	Some individuals stated that they already had the knowledge that related to information provided on the first steps program and that this knowledge remained unchanged. This implied that physical activity levels were unchanged and they were affirmed in the past choices they made. Five individuals (FS5, FS6, FS16, FS17, FS26) identified that the First Steps program affirmed their choice but lead to no change in behavior. For instance, FS5 stated: "*I mean it [physical activity] is not something that you need to stop doing.*" For FS16 and FS17 the PD had not progressed so no changes were needed and FS26 stated: "*as much as possible I forget about PD[therefore carry on as before]*". For FS6 the same barriers to exercises existed after the course no changes were perceived possible.<br><br>Summary Units<br>Decided already to up exercise FS5 Physical activity is something you don't need to stop doing FS5<br>Good to do, but nothing I didn't already know FS6 It would have been useful at diagnosis FS6<br>No changes in life FS16<br>I should do more but haven't changed activity FS17<br>I am positive about things, as much as possible I forget about PD. I take my tablets and get on with my life FS26<br>Continue as normal FS5<br><br>Verbatim Quotes<br>AS: Did it [the first steps programme] inspire you to do more exercises?<br>FS5: I had already decided that I would up my regime of exercise anyway<br>AS: in terms of the exercise content and impact? Did that have much value for you?<br>FS6: as it turns out I have been doing, PD warrior for a month now, it was good to do it, but it wasn't anything I didn't know already.<br>AS: and in terms of getting on with your life and content? Has that meant any different changes in your life?<br>FS16: no<br>AS: has it helped you, get on with your life or have other benefits in your life<br>FS6: not really<br>AS: did that result in your changing anything afterwards?

		FS17: not really, I probably should do exercise and things, but umm, I haven't changed much, no, I would say the Parkinson's has slightly got worse<br>AS: in terms of the exercise content and impact? Did that have much value for you?<br>FS6: that would have definitely had value at the beginning, at diagnosis, as it turns out I have been doing, PD warrior for a month now, it was good to do it, but it wasn't anything I didn't know already.<br>AS: if it had an impact on your health, where would you say that was in terms of after the program?<br>FS16: the same, I'd be very philosophical about it [about the PD], so you know, it is what it is and I am just dealing with it,<br>AS: any negative impact from attending the programme.<br>FS26: the answer to that is no, I am pretty positive about things but as much as possible I forget about it. I take my tablets I lay them out on the table each day. But apart from that I get on with my life.<br>FS5: I mean it [physical activity] is not something that you need to stop doing<br>AS: and in terms of the motivational content, did you feel inspired to do anything after the programme?<br>FS16: no, well I am not on any drugs yet, so that is the next step, to determine whether I am on any drugs yet<br>FS6:my biggest challenge is to find time to do the exercises I want to do and that remains the same
	The validated Narrative validation and affirmation. The beginning of impact and creation of change. I am doing the right thing Reinforced the need to do exercise. I could make a few changes too	A second related narrative told by participants illustrating impact was the validated narrative. This narrative identified that that First Steps program had helped them understand their past choices and thoughts were validated by the First Steps programme and that they were more motivated to continue physical activity or it provided a reinforcement of the message that physical activity had to be part of their lives with PD and they needed to engage in a greater way with it including 'stronger' exercise or increase what they were doing. The validated narrative was identified in 7 individuals (FS2, FS16, FS8, FS24, FS26, FS32, FS36). For instance, for FS36 it validated the choices made; "*FS36: I think it validated what I felt I should be doing…For instance, if I don't do that [exercise], I won't be able to do the other stuff anyway. It is about knowing you're not being selfish when you look after yourself*". Alternatively, FS8 identified the reinforcement of the positive benefits that physical activity could bring by stated "*I think I have always felt better when I have done exercise classes*". This narrative could also include statements that enabled greater engagement with physical activity. Individuals identified small changes learnt from the course that allowed this. For instance, knowing limits regarding physical activity participation (FS2), having a better understanding of the choice and technique of specific exercises (FS26).<br><br>Summary units

		Would already push myself quite hard, it taught me to know my limits FS2<br>Reinforces what I new FS16<br>A lot of it was what I was doing to a degree….some exercises they showed you could do yourself FS8<br>I realized I needed more personal involvement (to aid choice or technique of exercise) FS26<br>FS24: I try to be as active as possible<br><br>Verbatim Quotes<br>AS: good, in terms of limits of physical activity, has that changed in terms of what you can do or what you will attempt?<br>FS2: not really, I think I was, I always tired to push myself quite hard and the course was, part of the course content was not being afraid to say that you know your own limitations and I am tired and I am just not going to do this because I am too tired.<br>FS5: well I think, I have decided, continue as normal, making allowances for the medication to be taken.<br>AS: and would you say that getting that information helped you at all decide to change your behavior?<br>FS16: it reinforces what I knew and desire to achieve.<br>AS: thank you, in terms of the type of exercise, is it doing what you used to do, or is there any variation in that.<br>FS8: umm, well a lot of it is purely and simply, sort of, what I was doing anyway to a degree. But also there are some exercises that um, but they showed you, that you could do yourself you know. Sort of quite good to try and do well.<br>S: just out of interest in terms of the change in intensity of the exercise you are doing, after exercise, you feel better or any benefit from that post exercise, given you have increased the intensity?<br>FS8: no, I mean, I think I have always felt better when I have done exercise classes, for a long time, so I wouldn't say there is much change there because I have always felt much better for doing the exercise.<br>FS26: I was inspired by the physiotherapist because she was very practical and we did 10 different stations and whilst I was on those stations I realised that when I was at [name of health club] [name of place] I knew I needed more personal involvement and to go on equipment that would be more suitable for my requirements and that I found<br>AS: has it motivated you to do more and changed behaviour<br>FS26: the fact that there is somebody I can phone and ask can you come and give me another session I am not sure if what I am doing is right. It is a more personal, I now have someone like I did this morning that can come and look at what I am doing. Exercise is important and I am determined to keep myself.<br>AS: is there anything particular that you thought, oh yeah, that is really great, I'm going to start doing that now?

		FS8: not really, I think, sort of from my point of view, I, I'm, to me, I the point I am with it [PD], it is not something that figures in my mind greatly, other than I take the tablets three times a day, everything else I just carry on and deal with everything in the way I have always have done.<br>AS: have you changed your behaviour in anyway because of that?<br>FS24: i try to be as active as possible, as well as the exercises I do other things like i play bridge, i swim and that sought of thing<br>FS32: I think there is, I think from a umm, a perspective of being, you come in to contact with people of all stages and of all levels and some of those experiences can be quite daunting. But people that are on a similar level and have similar umm, attitudes towards Parkinson's, that can be very rewarding when you talk to them the people that you know are doing similar things to what you are doing as a individual.<br>AS: and did that way of thinking and choices in terms of exercise, was that something that shifted or helped you live in a different way?<br><br>AS: would you say it had a positive impact on your motivation to do more?<br>FS36: I think it validated what I felt I should be doing… But you think to yourself you should be doing other stuff, but what it has made me realise is the importance of that. For instance, if I don't do that, I wont be able to do the other stuff anyway. It is about knowing your not being selfish when you look after yourself because that is important. Because otherwise you cant carry on and do the stuff anyway. So I think that is what it made me realise, so by giving over a certain amount of time per week to exercise, looking after myself is not a selfish thing, it's a good thing to do, because the opposite would be selfish that would be allowing myself to decline faster and faster.
	The transformed narrative:<br>*The course or something or someone at the course paved the way for something new<br>*I have adopt the activated story – embraced limitations, gained hope, positive experiences<br>*I have got direction and I am putting it in	The transformed narrative revolved around a subsequent change of physical activity or exercise. The change was attributed to the course and related to an increase in the level, intensity or type of physical activity or exercise engaged in. This was directly attributed to the First Steps Course. This was identified by 8 participants (FS1, FS2, FS5, FS8, FS14, FS18, FS19, FS35). The changes identified included beginning a new class like Zumba Gold or Pilates (FS1, FS2, FS5, FS19), joining a gym (FS18), doing more walking or walking better (FS1, FS14, FS19) and changing a fitness regime or doing more in general (FS8, FS18, FS26, FS35).<br><br><br>Identified changes<br><br>FS1: I am getting more exercise because I was introduced to a yoga class<br>FS2: introduced to Zumba gold and I am doing that<br>FS2: PD warrior class didn't appeal<br>FS5: because of the class I have joined a group<br>FS19: started Pilates

	to action and gained mastery experiences	Fs18: joined a gym and enrolled on an online course<br><br>FS1: more walking<br>FS14: walking better<br>FS19: more active and walking longer<br><br>FS26: changed my fitness regime<br>FS8: encouraged me to do more<br>FS18: got an exercise bike and using it<br><br>FS1: umm, well, I suppose from meeting someone on the course another participant, I was introduced to a yoga class twice a week…so I am getting more exercise mainly because of this yoga class I was introduced to. Regards the exercise on the course, I must confess I have not, not being doing those.<br>FS2: so for example, one lady on the course told me about Zumba gold which I had never heard of, so I went off and investigated and started doing that.<br>FS2: umm, well we participated in the PD warrior class, but I haven't done anything like that since as it didn't really appeal but as I say there was one other participant who mentioned Zumba Gold which I have never heard of and having been looking around for something which appealed to me as a regular form of exercise, I went off and tried that and liked it<br>FS5: well the main benefit I have got from the group is to join a group in [name of place] and a group in [name of place],<br>AS: So you got a lot out of hearing and seeing those individiauls<br>FS5: yes,<br>AS: and them sharing was that the main benefit?<br>FS5: yes because as I say, there are things I didn't realise about, seeing the nurse, seeing the Parkinson's nurse on a regular basis. Talking, things to talk about, whether you take medication before a meal and all sorts of things. Small things, which one wouldn't really think about until, perhaps think about until you're away. In a way, it was good that we could share the information in a group.<br>AS: ok, in terms of the intervention and what the intervention has meant for your life can you say what changes?<br>FS18: umm, its given me the confidence to try and do more things, like I have joined a gym and I have enrolled on a online course to do some learning, I don't want to just stagnate. Whereas before I was sort of like, feeling sorry for myself I suppose. I have a positive attitude.<br>AS: Super, so in terms of the content, did the exercise content or the aspects which related to exercise have an impact on you?

		FS19: oh yes, I started Pilates since<br>AS: and what sort of motivated you to do that?<br>FS19: It was [name of person], [name of person] and the other girl as well. I mean everyone was able to reflect the positive benefit of exercise. I am been looking for a course for quite a few months, I have been diagnosed about a year, I have been looking for a course for a long time. It was the final piece of motivation when they said yes don't be afraid to go and try it. Feedback from the trainers to be honest.<br>AS: If you had to say what impact it has had on your life now, what would you say?<br>FS19: being part of a course that has allowed me to participate has giving me confidence with my Parkinson's and I am heavily involved with as much research as I can get my hands on really and also I am taking a few steps to start a peer group in the local area. I have always been the more I know the less I am going to be worried by it.<br>AS: so improvements transfer to others aspects of your life?<br>FS19: yes the improvement in strength have transferred to other aspects of your driving and the things that are naturally should come to you a bit more naturally.<br>FS26: one of the things that stood out and certainly made me change my fitness regime was, umm, at the end of the session at the end of second session we were introduced to a young lady who was a physiotherapists and who had specialised for the last 14 years with Parkinson's, for her clients. it transpired that she was made redundant by the NHS and she was going out on her own, or was going out on her own, so one could actually hire her for whole sessions or at home sessions<br>FS8: I mean it has encouraged me to do more anyway.<br>AS: is there anyone else you have talked to that has been useful to you regarding physical activity levels? FS1: umm, not really, apart from the yoga teacher I would say.<br>FS1: putting into practice what I have been learning I think and then the subsequent yoga classes, which were a direct result of being introduced to the Parkinson's group through the course. That was luck that I happened to meet somebody from the same town as me who was on the course<br>FS5: thinking about what they have said at the steps program about linking up, doing proper exercise , making the exercise being, well its no good just going to the gym and riding a bike for two hours, umm, over all it was a good experience, that is all I can think to add. It was something I was very pleased to be given the chance.<br><br>AS: so in terms of managing your Parkinson's what would you say the course has done for that?<br>FS18: it has opened my eyes up to see how useful Parkinson's UK is, when I do have a problem, I do and have contacted my local advisor. Umm, and yeah and the raising the awareness for the family, because I don't think when you are dealing with your own member of your family and it is sort of like more scary and understanding just goes out the window. Whereas if you go to an event like that when people have Parkinson's, have different stories to tell, it becomes less scary and umm, it gives them a more positive look

		for the future. It's sort of like the end of the world has come [for the carer, family or support person after diagnosis] and it is not the end of the world, its just a change of attitude.<br><br>FS1: I am doing more walking I would say.<br>FS1: since joining the yoga class I have joined several more and participated in one social activity so far, apart from the yoga class, so umm, it has lead on to other things.<br>FS2: Yes, I got of my bottom and went and did some exercise.<br>AS: and in terms of physical activity as part of your life before first steps and after first steps what would you say was the change if there was one?<br>FS2: probably a bit more of a nudge to make me realise that I needed to do things.<br>FS8: The exercises I did with the first steps were interesting and it was interesting because, the, the sort of description of sort of doing strong exercise, umm, which was good and in fact, in my other exercise classes I now push the effort, because I know that is what you need to do because sort of,<br>FS8: yeah, yes, that is what I mean. I think because of the Parkinson's your not consciously, but unconsciously not pushing, or the fact that, that is the nature of how it is. Where as the fact that now that I have done the sort of PD exercises, like the PD warrior pushing and sort of, so in your brain you are doing vigorous, so sort of like making sharper and pushing and I think that gave me a good insight into that side of things.<br>FS8: I mean it has encouraged me to do more anyway. Sort of, you know, sort of make sure you do a lot of exercise, but also when I am doing the exercise that I actually, sort of push the effort<br>FS14: I'm walking better, and when I do I am mindful to try and make it count and that kind of thing.<br>AS: would you say like you are more active now, compared to when you first started the exercise?<br>FS19: I am more active now, I was walking the dog for a long way, so after the first course, I recognized the benefits of exercising vigorously. I have cut the walking time down for the same distance. So all those sort of things motivated me as well.<br>FS19: umm, no, I think in terms of some of the negatives, some of the sessions weren't long enough in terms of the time constraints. But we are all going to have a reunion later in the year, so perhaps we can continue some of the conversations we started. But we had to truncate because of time constraints really. So there wasn't enough time in the day really. So it's a positive negative.<br>AS: In terms of your physical activity now, verses before the program, has that shifted or changed at all?<br>Fs18: considerably,<br>AS: could you expand on that?<br>FS18: I have got an exercise bike that I use, not as much as I should but I am using it quite regularly, umm, my son has decided he is going to do a long walk, he is going to walk a marathon, to fund raise for Parkinson's, umm, specifically aiming at first steps, because he found it so useful and he's got me training to do it, not the whole thing, because he is doing a 100k, but he's having me do the last 20 [k]. with him so, I am having to do quite a lot more exercise, but at the same time, I am feeling the benefits of it

		FS35: it was something I was already doing, I had read a little bit around exercise that it can make measurable differences. So I had made a concerted effort to be more active and not just. Because I suppose, one of the inclinations was to sit and think I cant do anything anymore and fade away. So I had made a decision to do more exercise, umm, some of that, I have not been able to maintain for various reasons, not Parkinsons. But it has opened me up to being more proactive about exercise, yeah. It was just good to be reassured that it can make some different.
**Theme 2; Internal mechanisms . Subtheme: Impact on control, hope and possibility**	*Take control, action and hope*<br><br>*focusing forward and hope<br>*I appreciate the story and could adopt it – adoption mean accepting and embracing limitations, allowing hope, and generating positive emotions<br>*being introduced to something that is appealing, a reason to be activated	This theme was generated as a result of participants seeing that a future was possible, that action could be taken as illustrated by others who are sharing their own experience. The theme was supported by 9 participants (FS1, FS2, FS8, FS14, FS32, FS35, FS36 and FS38). The theme was illustrated by the recognition of the importance of physical activity and exercise in maintain physical well-being. For instance, FS32 stated; *"if you as a Parkinson's patient want to keep active, want to keep your mobility, balance and things of that nature, you have to, you have to put in the effort and do the exercise*." FS14 summarises the theme and the importance of taking control and action to enable living with PD: "*AS: in terms of getting on with your life? What impact would it [the First Steps programme] have had on that? Any change? FS14: well, I, I think, what was important for me at that stage was that you could actually, fight back to some degree. Take the future in your own hand to some degree and that's is quite important*." This theme was a key illustration of the impact of the stories shared by participants and outcome of social comparisons and the ability to relate to others and take onboard the message to utilize it in their own life situation.<br><br><br>FS2: Plan to action…identification of what will be done<br>FS14: fight back to some degree…take future into your hand<br>FS1: seeing what the presenters were managing to do<br>FS5: recognition that I can do what was presented an I intend to…see what can be done in 5,10 years time<br>Fs8: interesting to see that other people have it and to see peoples perceptions<br>FS32: I think the first steps course, was valuable, in as much as a lot of the information that was umm, given out, was stuff that you don't know as a patient, as a newly diagnosed patient. So it has benefit to form your expectations of what sort of, lies ahead if you like.<br>AS: and in terms of changing your behavior or doing things differently after the program?<br>FS32: yeah, yeah, I think the exercise was, the most, the biggest thing for me, was to keep a level of daily exercise going and that is what came across from the contact. That there is, if you as a Parkinson's patient want to keep active, want to keep your mobility, balance and things of that nature, you have to, you have to put in the effort and do the exercise.

		FS32: the key value for me is just emphasizing the fact that exercise is key and a positive mind set and trying to take control of your own issues, trying to take control is, umm, the best way I can describe it anyway.<br>AS: just summarizing the impact on mood or mental well-being what would you say?<br>FS32: I think I would say, that it, gives you, a first, the course is called first steps and that is very appropriate because it does give you a first step into finding out about this disease you have been diagnosed with, and I think if you, if your minded that that is what it is and that you can carry on your own gathering of information and knowledge. That is what it is, it is a platform for you to think I am going to go forward with this and try and deal with it in a way that you feel that is most appropriate for you.<br>FS35: So in terms of confidence of seeing others, I work in a hospice, so in terms me, I was seeing Parkinsons at the end of life, so I was seeing people much further down the road, so it was important to see people much nearer diagnosis, so it helped having it normalized.<br>FS36: I think meeting other people and seeing that it is not the end of everything. I think the leaders were very good at that and just thinking that although it is not going to go away you can live with it very well and umm, try and mitigate some of it by life choices that you make like exercise and things like that.<br>FS35: I think it was a sense of empowering really. You don't have to sit away in a corner and fade away. Because you are living with this diagnosis and you are not sure what it means. I mean the Parkinson's UK website is really good but there is limited amount of information that it can give and there is only so much time that other people have, you know, practitioners that can talk to you, so actually having someone that you can actually have it reported to you is needed and it is good for yourself and it shows you that you can live a fuller life it may be that it is just a bit different to the one you were thinking about.<br>AS: and meeting these other people, umm, that was useful because you could relate or get insight? What were the benefits if you had to pin point<br>FS38: One of the key benefits is that Parkinson's is a very broad term and its impact on different people can be quite different. In terms of physical symptoms and in terms of the way in which they respond to being told they have got this condition. Umm, and I think as a result I am much more positive in terms of looking forward and my outlook in terms of what may happen in the future. So really positive.<br>FS38: I mean, on the second day of the course there was quite a lot of practical information and feedback which was very helpful.
**Theme 2; Internal mechanisms. Subtheme: Positive impact on mind-set**		Many individuals talked about the impact on mood and mindset from the program, identifying a protective mind-set against the experiences of the symptoms and an acceptance of the feelings or emotions about having PD. For instance, FS18 stated "*I don't want to just stagnate. Whereas before I was sort of like, feeling sorry for myself I suppose. I have a positive attitude*." This theme was identified by 11 individuals (FS1, FS2, FS6, FS13, FS14, FS18, FS19, FS24, FS32, FS36; FS38). The impact on an individual's mind-set had several influences. This included thoughts about the programme before attending because of the opportunity to give and share with others (FS2), the value of meeting varied people with different outlook (FS38). Further

		individuals highlighted a positive atmosphere within the group (FS1, FS18). This was identified as important because of the worry (FS24), difficulty (FS19) and sadness (FS14) that can come with diagnosis of PD. FS13 noted that the content of the session could be depressing. FS13 only attended the first session and it was noted by another participant that the experience of the second session could be more positive; "*people [who were] negative on first sessions more positive on next session*" (FS6). This theme linked to hope because it related the impact the course had on psychological adaptation and emotions.<br><br>FS1: mood was raised<br>FS1: positive general atmosphere<br>FS2: knowing it will be giving and sharing you go along in a very positive form of mind<br>FS6: transformation of others – people negative on first sessions more positive on next session<br>FS14: programmed designed to cheer people up a little, not the end of their life<br>FS18: nice to be positive<br>FS18: I have a more positive attitude because of the group<br>FS24: being diagnosed with PD is alarming to deal with<br>FS19: well-being has improved and it is important to realise it is not just you that finds difficulty and not always Parkinson's to blame<br>FS14: reassurance because it is not a happy disease to have<br>AS: was there any changes as a result of that?<br>FS32: yes, I think, motivation I think is something that, you know, I do have a high level of motivation and the information that came out of the group sessions, was, umm, positive, in you know, very applicable to my particular mindset.<br>AS: impact on mood – would you say there has been a positive impact?<br>FS36: it is difficult to answer, because people have good days and bad days, because you have a period when you are doing well then some time when you are not. But it has made me maintained a more positive mood, because even if it feels like you have had a bad few days it perhaps doesn't get me down as much as it might have done at one stage. I think it is accepting that it is probably going to happen and not, not just succumbing to it and being aware that, that is going to happen and that is what it is.

		FS13: rather depressing (the content of sessions)….but interesting (person only attended 1 session)<br>AS: and meeting these other people, umm, that was useful because you could relate or get insight? What were the benefits if you had to pin point<br>FS38: One of the key benefits is that Parkinson's is a very broad term and its impact on different people can be quite different. In terms of physical symptoms and in terms of the way in which they respond to being told they have got this condition. Umm, and I think as a result I am much more positive in terms of looking forward and my outlook in terms of what may happen in the future. So really positive.<br>FS38: But in terms of the general overall outlook has been influenced positively.<br>FS38: no I think generally it has helped in the process of becoming more positive of about life and the future but that has been part of a number of things that have helped in that direction.<br><br><br>Verbatim Quotes<br>AS: and in terms of impact on mood or anxiety is there any, do you recall any impact on that?<br>FS1: my mood was raise for the weeks afterwards. It increased my mood. It increased my mood at the time<br><br>FS2: funny enough on the first steps, umm, the first, we got a print out saying five things I am going to do by, you know by the end of the course and I wrote myself a promise to take the stairs two at a time, so I was always doing stretching<br>AS: in terms of getting on with you life? What impact would it have had on that? Any change?<br>FS14: well, I, I think, what was important for me at that stage was that you could actually, fight back to some degree. Take the future in your own hand to some degree and that's is quite important.<br>AS: is there anything else from the content program that has motivated you to change what you are doing?<br>FS1: it was a while ago now, umm, yeah there was, umm, there was a general atmosphere of positive general attitude and having two presenters, also with the condition and seeing what they manging to do, with the balls, and cope with the organisational challenges.<br>AS: ok, that's interesting so there is definitely a value in sharing that information, on a certain level, or in a certain forum, which may be research focused around sort of around positive psychology almost, its, positive coping<br><br>FS2: yes I think definitely, and I think because you do to something like that. Knowing that this is what it is about, the whole day is going to be giving and sharing and you go along in a very positive form of mind so, I would say that forum is brilliant but nothing since then.<br>FS5: I suppose coming away from the steps course, it made me think, well, I can do that and I can do this and I am going to and that is just the way I am living my life at the moment.

AS: so meeting particular individuals that have good experience to share, that has had a lot of value in terms of what could be done?
FS5: yes, what could be done and how you can be, 5, 10 year's time, you know if you look after yourself.
AS: in terms of any negative impact that you have found on the program? Have you found anything?
FS6: no, that wasn't a problem, there was a few people on the program that were quite negative on the first sessions. But they were, more positive on the second session, so it really did something for them and that was good to see.

AS: and in terms of experiences of, did you hear anyone else's stories, or anything from others that inspired you
FS8: I think it was good, it was interesting to see that some people had a similar experience of discovering that you have got it and it was interesting to see people's different perceptions of how they dealt with it.
AS: so do you think that the advantage of that, and seeing the range of people and experiences, how would you summarize the benefit of that to someone else who was thinking about doing something like that?
FS8: Well, I think it was, just reassuring, I suppose in a way, it was the sort of, know where you were, I don't know, it was valuable, but it would be difficult to sort of say, you know, you know I certainly would say that it was good, it was something that I was glad I did, umm
AS: in terms of the content and what was delivered was there anything about the content that you thought that was really good or not so good
FS14: I thought I did some sort of assessment at the time, in terms of content, what it covered, it covered really well in a sense I thought, it was a program designed to cheer people up a little bit, to understand that it wasn't necessarily the immediate end of their life.
FS14: I think I took, some comfort in the realization that some people had this disease with them for a long time and remain functioning which is what my great fear was, when I was first diagnosed.
FS14: yeah, no, I think it was timely, I think I needed some sort of support, I think whatever happens you have to come to terms with that, but you have to know what the parameters are [what is possible, how do people live, what is beneficial]
AS: would you say it has left you more confidence following the course?
FS18: yes
AS: what impact would you say there has been on your mood and anxiety?
FS18: what from the course?
AS: yeah,
FS18: umm, I think it is nice to be positive
AS: and as a result of that have you found other benefits?

		FS19: Well, I think my sense of well-being has improved and I've answered some of those nagging questions, which you can only sort of tease out by being in a group of fellow suffers you sort of can talk it though and realise it is not just you and it is not always the Parkinson's disease to blame .<br>AS: couple of questions on confidence, on mental factors?<br>FS19: for me it has improved and it is funny, in my feedback as soon as I finished the course I wrote a little note to, umm, I think to [name of person] actually, which was that when I went into the room I could see a difference in all the delegates faces which I am sure they could see in mine and you could see from the anxious and unknown from the first course and perhaps the unknown about the day and the unknown about the condition. I think some people have gone away and investigated various avenues of benefit of exercise and all the things which were troubling them and everybody came back and you could see the sunshine [with them]. A lot of people who didn't come back, which is a shame, but of the people who did come back there was sunshine in their faces, that is for sure.<br>AS: is there anything you would like to say regarding the first steps experience:<br>FS24: I just think it was terrific, when you have been diagnosed with Parkinson's it is a bit alarming to deal with it and to have information to hand like that I think is fantastic. It was one of my colleagues at [name of place] who strongly recommended attending. though him. and actually the Parkinson's nurse at [name of place]<br>AS: and was there anything particular about that, that stood out to you? Or had personal benefit<br>FS8: I think it gave you, umm, err, a better sort of awareness, of what was there for you.<br>FS14: yes, it is reassuring, but I looked for more than reassurance, umm, because it's not a happy disease to have. You look around at people who have it for a while and sometimes its rather sad and you think, I'm, I'm on that track [an understanding of what will or could happen in the future], umm, but not necessarily so. I am interested to know more about the development [of Parkinson's]. I have got to say, there has been much more support than I expected and its been worth while.<br><br>FS2: And also one of the presenters made it quite clear that whatever stage you are at, you should never be afraid to put your hand up and say I can't do this because I have Parkinson's.<br>FS18: umm, it's given me the confidence to try and do more things, like I have joined a gym and I have enrolled on a online course to do some learning,<br>FS1: oh yes, seeing these two presenters and how much they were achieving and umm, also talking to the other people on the course and umm, yes, it did increase my confidence yes, also they talked about foreign holidays and that was err, quite confidence boosting.<br>FS2: oh, absolutely, as a health care professional retired, I was very much an advocate for people in my care, but not very good at standing up for me. So first steps gave me the confidence to say, excuse me, I have got Parkinson's.

		FS14: I think probably I am more comfortable in social situations, for instance, going out to dinner with people. Certainly, I used to think I would be the center of attention and wreak every bodies night.<br>AS: did the course impact that? Or some of the teaching in terms of the social confidence?<br>FS14: I think you could see people who had the disease who are managing in that way. So that was very important.<br><br>FS18: I don't want to just stagnate. Whereas before I was sort of like, feeling sorry for myself I suppose. I have a positive attitude.
**Theme 2; Internal mechanisms . Subtheme: Impact on confidence**		An increase in self-efficacy and being confidence about living with PD and illustrated by how others achieve that. This was identified by six individuals (FS1, FS2, FS14, FS18, FS36, FS37). This included being aware and sharing with others your own needs because of the PD for instance FS2 stated: "*one of the presenters made it quite clear that whatever stage you are at, you should never be afraid to put your hand up and say I can't do this because I have Parkinson's*". Speak up about, second to this individuals identified the confidence to do more activities and interaction including the confidence to enroll on a course and join the gym (FS18), the confidence to attend a dinner party without worry about wreaking other people's night (FS14). This theme was the likely result of successful engagement in physical activity following the program which had a direct influence on an individual self-efficacy.<br><br>FS2: presenters made it clear you need to speak up about PD<br>FS18: confidence to try more things, I've joined a gym and enrolled on an online course<br>FS1: what they talked about was confidence boosting – making things possible<br>FS14: going into social situation and not worrying<br>AS: would you say it has given you confidence to what you wouldn't have done before?<br>FS36: I think yes, because before I was diagnosis and at first I had lost a lot of confidence, I had given up what I was doing, my job because I couldn't carry on at the time. Now I am diagnosed and having treatment I am probably better than I was 2 years ago. Now I am retired and I have now taken on another job and not I am talking with my employer that is more complex. So I think in a sense it has given me confidence that I can do more than I was thinking I could do.
**Identified impact on others**		*Individuals identified the importance of the programme for the spouse, family member or friend who attended the First Steps programme. This theme was identified in seven individuals (FS16, FS18, FS19, FS32, FS36, FS38). The benefit appeared to be in the ability to relate to others for instance* FS36 stated: "*I think my husband found it useful hearing other spouses experiences and their frustrations*."

		For instance, it was no<br>*Understanding for others - impact*<br>FS16: benefit for wife to aid understanding<br>FS18: benefit for husband to understand it<br>FS19: benefit to wife to understand what it is and what it is not<br>FS19: see what it is and what it is not<br>FS32: I think it gives the patient and in my case my wife, who is a massive support for me, it gives you both a greater awareness of the disease and how people deal with it.<br>FS32: think, my wife took a positive role in the course as well and I think she gained knowledge from talking with, with the other partners or careers that were on the course and particularly from [name of course leader] I think she was able to relate to a lot of information that he was giving.<br>AS: was there any impact on the wider family?<br>FS35: no, no, I kind of support them. Not the other way round.<br>Fs36: I think my husband found it useful hearing other spouses experiences and their frustrations.<br>FS38: my wife also found the experience very helpful as well meeting others who are living with partners or know friends who have Parkinson's.<br>FS38: It was a very positive experience for both of us and perhaps me in particular. I cant think of a single negative thing about it.<br>AS: you mentioned your wife attended with you and there was benefit, what was that benefit?<br>FS38: it is very much understanding and you know it is just helpful to talk to someone who is living with someone else who has parkinsons in terms of what difference that makes and any particular tips that they may have to offer.

Psychosocial mechanisms explaining impact

**Theme**	**Definition**	**Example Unit**
**Social comparison**	Definition: I can relate to or I am similar to	An essential mechanism explaining the impact identified above was through social comparison. Most frequently this was identified as being able to relate to another individual who has had a similar experience. The First Steps programme was designed around an ability to allow this through stories which show a positive and possible way to live with PD. Within each day there was also an opportunity to talk with others, listen and understand others stories. This

	the other people here. What they say and how the approach the illness is similar to how I want to approach the illness.	theme was supported by 14 individuals (FS1, FS2, FS5, FS7, FS13, FS14, FS18, FS19, FS24, FS26, FS32, FS35, FS36, FS38). Most comments related to the benefit of understanding others experience and broadening one's own knowledge of PD. For instance, FS17 stated: *"it is interesting for me to see other people, you know how different we all are"*. This process primarily allowed individuals an insight to how others coped and manged the condition and a chance understanding how others viewed or let the PD affect them. This theme was captured by FS2 who summarised the value by stating:<br><br>"it was from listening to the presenters who have had Parkinson's for such a long time, umm, and how, how severe they have got it and how well they have coped with it and their coping mechanisms, made me realise, I am at the beginning of the journey that they are already on, umm, and they are obviously very wise because they have learnt a lot about their disease and management and umm, you know all sorts of coping strategies, both psychological and physical and I need to listen and take them on-board."<br><br>There were two instances (FS26, FS18) where this was identified as not so beneficial; FS26 identified that the group appeared '*concerned about giving away their own position*' in this group it was perceived that the benefits of sharing was not occurring. Alternatively, another participant (FS18) noted that one lady was reluctant to embrace ideas.<br><br><br>FS1: inspiration to meet people with same condition and see how they were coping<br>FS2: good to talk to others and see how they were coping, good to listen to presenters and identify how they are coping with PD being worse<br>FS7: comparing notes was valuable<br>FS5: seen people at another group, you can either let it affect you or do something about it<br>FS14: interesting to look and compare with people who have had it for a while<br>FS17: interesting to see other people and how they are coping<br>FS19: condition of group and empathy and understanding<br>FS24: hearing form others because the ordinary person doesn't know what it is like to live with PD<br>FS26: grouped appeared concerned about giving away their own position<br>FS18: another lady was reluctant to embrace ideas<br>FS13: Interesting to seehow others are dealing with PD<br>FS32: …the information that came out of the group sessions, was, umm, positive, in you know, very applicable to my particular mindset.<br>AS: so you could relate to others?<br>FS32: yes, I found that to be, my expectation was that it wasn't going to be quite so much umm, in that direction, so I was pleasantly surprised, it fitted in with my, the way that I am treating my condition.

		FS32: the word relatedness is the word that would describe it best, we both have an understanding and can relate to the experiences of others.<br>FS35: it was just the appreciation that, there were other people of a similar age to me, on that kind of first part of the journey, of coming to terms with Parkinson's.<br>FS35: I am pretty much on my own here, I mean the Parkinson's nurse has got 600 patients, people much further down the road with their parkinsons and a huge advantage of the FirsSteps course was meeting people that were at the same place as me. I don't need to see somebody much worse than me because it feels like having my nose rubbed in it.<br>AS: so you got benefit out of the course, as a result of that have you looked for a group?<br>FS35: no, again there is the same problem, I do go to a Parkinsons exercise class in [name of place] and I'm 20-30 years younger than all of the people in the room. I don't need that thank you.<br>FS35: I felt having two men run the course was slightly off kilter. you need the compliment of a man and a woman because we relate differently<br>fS35: probably improved my confidence of seeing other people, 12, 18 months down the road from diagnosis, they are doing ok, and we are all doing ok. that helped a lot.<br>AS: what was the benefit in meeting other people<br>FS36: I think disappointingly the people on it were not our sort of age. They were older and retired. So obviously there priorities and experiences were a bit different. Then saying that they were not old and doddery, they were a decade older than us. It would have been nicer if there were more people of working age.<br>FS36: So the course was useful because it explained what things are available and how you can access various things. I think it was also useful because it made me realise the importance of research more, so I have been an eye on what has been going around in terms of research and taking part in anything I could do and just knowing your not on your own. Because for a long time I felt very much on my own, because there was nobody else you know who is going through this. Umm, then to be in, where there is a group of people, although each person experience is different, at least there is some connectedness. Some understanding of what people are going thorugh.<br>FS36: I think it was important that the people running the course also had Parkinson's and it was also important that umm, so both had had it for some time. So it is good to see that people are still leading active and full lives even though or despite the progression of the disease, I know it progress differently for each of us, so I mean it was just nice to be able to vent a little bit, frustrations,<br>AS: is there anything negative about meeting other people?<br>FS35: I had a colleague when I was first having symptoms, and I had a coleage who was in his late 40s who went to a Parkinsons group and he said it was the worst thing he could do because he saw people so much further down the journey, he found it very hard to cope with and also a Parkinson's nurse so I remember that and there are two groups locally and I don't go to that one because they are much older and it's a different thing, but the other one is more of a working age and if you need to go to a group go to that one. So I could image that it would be possible to go to one of these courses and see people much further into the disease and the condition and being quite scared by it yeah, I suppose it is going to happen isn't it?

		FS38: The health care professional has a key role to play in my case the Parkinson's nurse who was really helpful and supportive person, but that cant replicate the experience of 6-8 other people.<br>FS38: I know a number of people who have Parkinson's. umm, and there attitudes vary considerably from perhaps wanting to get involved at a local level to actually not wanting to be reminded as you just said. About how that condition might progress, in terms of the first steps program we are talking about people who have been recently diagnosed so anyone on that course may have had good or bad experiences with people with Parkinsons who are perhaps several years into that situation. But meeting people on the course who were recently diagnosed didn't expose you to that concern.<br><br><br>FS1: that was quite inspirational, to meet people with the same condition and how they were coping,<br>FS2: umm, it was just good to talk to other people and see their ways of coping and err,<br>FS7: and just comparing notes shall we say.<br>FS1: It was drummed into us that exercise was, really beneficial and I would like to keep to that and do more.<br>FS1: that was quite inspirational, to meet people with the same condition and how they were coping, and umm<br>FS8: The exercises I did with the first steps were interesting and it was interesting because, the, the sort of description of sort of doing strong exercise, umm, which was good and in fact, in my other exercise classes I now push the effort, because I know that is what you need to do because sort of,<br>FS2: we both found it very helpful on the tutors very interesting, very interesting and the other people, in a similar position<br><br>FS2: yeah, I had not found anything that had worked for me, as I say one particular thing, one lady suggested and I went out and tired it [Zumba Gold]. Which was really useful.<br>AS: was it mainly physical activity related information that you found useful or was it other information that you found useful?<br><br>FS2: No, all sorts of information really, umm, I mean, do you want examples or?<br>AS: examples would be useful, what you felt you could take forward and use that information and its going to benefit me.<br><br><br>FS2: Well simple things like I had never heard of a radar key for a disabled toilet and within a week I had my radar key off the internet and so, it was just really useful.<br>FS2: I was busy as a labour ward mid-wife I was busy, anyway, but exercise doesn't appeal at all, so I would go to work come back and potter in my garden.<br>AS: ok, in terms of the intervention and what the intervention has meant for your life can you say what changes?

		AS :in terms of confidence, was there any impact on your confidence?<br>FS5: I mean I have joined a group, an exercise group down in [name of place]. There are people there who have had the Parkinson's for some time. So clearly, you can let it affect you, its seems to me, or you can carry on with your life.<br>FS2: It has made me more able to say, to my husband, family and friends, I cant do that because, or planning better, so, its not a great idea if I do that busy day followed by that busy day because I know I just wont cope with it and so its being honest and being able to say can we not do two days together, can we separate it with a rest day because I will be too tired. Whereas before I would just try to carry on like I had done for 20 years in the NHS.<br>FS2: yes, yes definitely, it was from listening to the presenters who have had Parkinson's for such a long time, umm, and how, how severe they have got it and how well they have coped with it and their coping mechanisms, made me realise, I am at the beginning of the journey that they are already on, umm, and they are obviously very wise because they have learnt a lot about their disease and management and umm, you know all sorts of coping strategies, both psychological and physical and I need to listen and take them on-board.<br>FS2: I am sure there were a lot of people who found that really interesting. But that's just different for different people. There are people who really really like Yoga and I just think, boring, umm, so I was prepared to try everything and listen to everything, but for me the one thing I did take away was that I am not going to do that type of exercise. So I need to find something else, which I did.<br>AS: yes, ok, ok that is really useful. In terms of the negative impact, is there anything negative from the course or that didn't work for you?<br>FS2: absolutely not, I enjoyed it all, just because I didn't take it all away and do absolutely everything. I still enjoyed it and we both felt that even though you know I had some medical knowledge as a midwife and my husband had a lot of medical knowledge as a doctor we still both benefited from it and were glad we had gone.<br>FS5: yes, I thought they were both very good and it was good to see someone [with PD]. One of the said, he had the Parkinson's for eleven years and you would have never of guessed it looking at him. So it was sort, it was useful in the sense that, you see the tremor and the badly affected people and to go there and see someone with Parkinson's, who has had it for eleven years, who has obviously go the right medication and everything, this was interesting and useful to those of us who attended the class.<br>FS5: so it has just given me a bit more confidence,<br>AS: is that more impacting than if a health care professional told you what is possible?<br>FS5: I think has to be a mixture of both, health care professionals give you some guidance but people find out these things and if you join a group for some exercise people actually make comments about what they are doing how they are doing it and if they are taking their medication at the wrong time of day. Things like that.<br><br>FS5: made me look, to see what was available [possibly in terms of medication], rather than just saying I will sit back and take the medication. Umm, it was clear from the two guys that run the program that you have to help yourself. But if you help yourself, it may make a big difference in the way you lived your life with the disease.

		AS: would you say it is worth taking part in the program?<br>FS17: yes, it is interesting for me to see other people, you know how different we all are.<br>AS: and what was it about the other people, was it there experiences and what they shared?<br>FS17: yes really, sort of talking about how they are coping with it.<br>AS: would you say that is the most useful thing for you?<br>FS17: yes, possibly, yes<br>AS: if you had a chance to recommend the course would you recommend it?<br>FS17: yes I would<br>AS: and what would be your reason?<br>Fs17: well the two that ran it actually had Parkinson's, which I thought was a good thing. For someone who doesn't have it to run the course, they don't know [what it is like] and I know we are all different. No, having someone who has actually got the same as you was really helpful.<br>AS: that is really useful, in terms of the, stories you heard from people did that have value?<br>FS18: Yes, it is nice to have the perspective of other people's way, there journey so to speak, umm, and I think for my husband's daughter in particular it was enlightening.<br>AS: is there any part of the course you put that down to?<br>FS18: that is a good question, I think the two guys that do the course are inspirational anyway, and the fact that they made it clear that you have Parkinson's it doesn't have you. Umm, and that it is not the end of the world and if you have a positive attitude you can keep living. But I cant say off the top of my head, if any particular part of the course is responsible.<br>AS: so would you say it has had a positive impact on your motivation?<br>FS18: yeah, I think, the one phrase that has stuck with me, is that it is alright to be selfish. Umm, yeah and the use it or lose it attitude. They both, sort of motivated us a lot.<br>AS: The competence of the staff and manor of the staff was that ok?<br>FS19: oh, no excellent, everybody was very kind, very knowledgeable very compassionate, very empathetic, and obviously being PD suffers as well, people with Parkinson's there was a lot of empathy and understanding between everybody I think.<br>FS19:That was another positive, about discussing each others medication and what it did for them and perhaps the reasons why and the generic drugs and problems with generic drugs, it was answering questions that you would not get an answer from anyone else on.<br>AS: how did you feel now compared to when you first started the programme?<br>FS24: I think the key to the first steps program was information, because the ordinary guy doesn't know anything about Parkinsons. So mentally in that respect it was encouraging and being able to hear other people talk, umm, whether I feel better, marginally i think, but it was the fact that you learn things like it is not life threatening and that was encouraging and hearing other people.

		AS: any impact on your family or when you volunteer or other activities:<br>FS24: I think the fact that my wife was a retired district nurse, she got benefit from it as well, she attended, so we have doubled up really<br>AS: and the benefits for your wife were the same as you?<br>FS24: absolutely<br>A: an in terms of hearing stories from others at first steps, did that have any value?<br>FS26: I think it would be if there was a follow up, a year on. I didn't find that, (a) because I think we were a little concerned about giving too much about our own personal position. In other words I am not as bad as him. Without actually going to the person and saying what are you like and what are your conditions? Like I say the only one that stood out was the one that unfortunately unless he got round to doing some strenuous exercises him, he got himself into a better place he was definitely not for this world for very long. He looked after two sessions with a physio, he really looked ill. He was the exception. I was also concerned from the boys, the first steps presenter on the second session there was only 4 people who turned up and on the first session there was 6, why I don't know.<br><br>FS1: yeah, also I went away from the course, with the knowledge that umm, exercise was key to mitigating the effects of the condition. That is the message I came away with yeah<br>FS1: umm, well, I suppose from meeting someone on the course another participant, I was introduced to a yoga class twice a week<br>AS: any impact on your mood or anxiety?<br>FS2: I didn't come away with, from the other participants, a blinding flash of inspiration but there was a sort of sharing of oh, yes, I understand that and you know. So it was, it was a comfortable environment…. the whole group were all at a similar stage, we were all receptive, very much to the presenters but for each others.<br>FS5: as I say there was also talk about when to take the medication. It hadn't really bothered me, but some people, a couple of the ladies there had tried taking the medication on an empty stomach half an hour before a meal or something and had felt unwell. So I thought, from I thought, well I haven't had that problem, but it was useful to know that people have different experiences,<br>AS: and was it worth taking part?<br>FS7: yes, yeah, yeah<br>AS: What is the main reason for saying yes then?<br>FS7: meeting other people in the same condition, umm, and just comparing notes shall we say. Umm, yeah so, it is just interesting to see other people.<br>AS: and that benefits you in what way?<br>FS7: How Parkinson's affects different people in different ways and how many different symptoms there are and that people had different symptoms. Like, some people have no sense of smell, well mine is fine, and, you know, my problem is the vivid dreams, so it is interesting to see that part of it.<br>AS: brilliant and in terms of the exercise content and impact of that was there any value of that?

		FS8: yes, that was very interesting<br>FS8: yes, but it made you aware that there are different sort of people there, umm, that were at different stages, although we were all fairly newly diagnosed, people were further along the line, umm, and you know, and I felt very confident with the sort of treatment that I had been having,<br>AS: in terms of the exercise content and focus around exercise, was that interesting?<br>FS16: yes that was interesting<br>AS: can you tell you me a bit more about that?<br>FS16: I think it was just bringing home the fact that exercise played an important part [in the management of PD]<br>AS: got a few questions about how you feel about it compared to when you first started the program?<br>FS26: a lot more knowledgeable regarding what Parkinson's is all about.<br><br>AS: last couple of question, any perceived negative impact, from the course?<br>FS18: not from the course, no, I think, umm, from some of the other participants may be.<br>AS: can you expand on that?<br>FS18: I think, there was, umm, I think the women in particular I am thinking off, was, she thought, she'd expected her Parkinson's was clear, it wasn't, she hadn't, so she was reluctant to embrace any of the ideas<br><br>AS: so there were some interesting outcomes as a result of the course, other stuff going on [in the group]?<br>FS2: yes there was, just people chatting and saying, well I have tried this and it was good, or I have heard of this and the questions people were asking were also questions you would have wanted to ask yourself. So it was good information sharing.<br><br>FS16: I would say it was good for my wife. Who I had obviously taken with me for an insight as to what to expect.<br>AS: would you say that was one of the best things about the course?<br>FS16: yes, that we jointly attended and then could be more open and discuss what's what about going forward.<br>FS18: I think for my husband's daughter in particular it was enlightening.<br>FS19: My wife attended the course and it done her a lot of good. Because she could see and understand you know, it is not the end of the world, it is part of a journey. So, and so that was the biggest plus for me it was helping her understand [about the PD].
**Social Control**	**I was given advice which I**	Social control was identified within the mechanisms as participants could identify direct instances of when advice was given to them and taken on-board and embraced. This was identified by 9 individuals (FS1, FS2, FS5, FS7, FS8, FS16, FS26, FS35, FS36). It was reflected by general statements regarding physical activity and exercise and undertaken more

	**agree with and have used that advice to change**	intense exercise and devoting more time to it. It also was reflected in specific comments that illustrated a change in physical activity or exercise like participating in a new Pilates or yoga class. For instance, FS2 stated: "*one lady suggested I went out and tried Zumba gold which was very helpful*". This them went further than just including physical activity as advice was also given about living with PD and having access to aspects which improve life like a radar key, or when to take medication. Some of this was summed up generally, for instance FS7 stated that the benefit was through "meeting people with the same condition and comparing notes, it is interesting". The benefit of this understanding was identified for people's partners also. Three individuals (FS16; FS18; FS19). This included the identification of benefit of understanding what PD was and what it meant. This understanding was associated with positive interactions following the course.<br><br><br>*Social control*<br>FS8: importance of doing strong exercise<br>FS2: one lady suggested I went out and tried Zumba gold which was very helpful<br>FS2: I'd never heard of a radar key<br>FS2: helped me be honest with others about limitations and planning<br>FS5: it is about information from peers about the lived experience<br>FS1: introduced to yoga class by someone on the course…came away thinking exercise is key to mitigating effects<br>FS1: inspiration to meet others with similar condition and how they were coping<br>FS2: good to talk to other people<br>FS7: meeting people with the same condition and comparing notes it is interesting<br>FS8: made you aware of different people there and different stages of the condition<br>FS16: interesting to see what benefit of exercise could be<br>FS26: more knowledgeable about Parkinson's<br>FS5: talk about when to take medication…useful to know different people's experiences<br>FS35: I cast out a statement at some point in the first one [session] and it was closed right down and I knew what was going on because I am pretty self-aware and I am thinking you can't cope with this can you.<br>FS36: it has also opened up, I go to a keep fit class that one of the people on the course runs who herself has got Parkinson's, so I discovered that and so, apart from that one person that is the only person I kept in touch with, [place] is a big county so people are Strone around a bit.
**Untold story**	**Opportunity to meet others not possible**	Six people (FS1, FS2, FS5, FS32, FS35, FS38) identified that they had not met others with PD before the course. This group identified that they would not have known the valuable information if it wasn't for the course. Another participant (FS6) identified needed the information earlier that received for the same reason. Importantly one

individual (FS2) identified that she would not do anything like the course again. That once was enough. Further one more participant (FS8) identified that they didn't want to share with others stating:

"*well, I made a conscious decision not to talk [about PD], other than sharing it with some immediate family members. I made a conscious decision not to say anything, on the wider side of things, because I know, of two or three people who had diagnosis of Parkinson's and also the reactions that people have given and my feeling, for me personally, was that, until the time comes when it is obvious enough that people need to know, I don't need to share that*"

FS1: before course hand met others with PD
FS2: if hadn't done it would known
FS5: wouldn't have known – gave me information to what I wasn't party to
Fs6: need it within weeks of diagnosis
FS8: didn't want to talk with others

FS1: well it was very interesting to meet other people with the same condition and I hadn't actually don't that before.
FS2: . because without having done it I wouldn't have known.
FS5: I didn't even know they existed until I started talking to other people and, taking the advice, thinking about what they have said at the steps program program?
FS5: oh, yes it gave me information that I wasn't party to, so I think I have learnt from it. So I think it is better, if that's the one word.
AS: was it worth taking part in the first steps
FS6: I think it would have been more useful within weeks of the diagnosis as opposed to being almost a year on.
FS8: well, I made a conscious decision not to talk [about PD], other than sharing it with some immediate family members. I made a conscious decision not to say anything, on the wider side of things, because I know, of two or three people who had diagnosis of Parkinson's and also the reactions that people have given and my feeling, for me personally, was that, until the time comes when it is obvious enough that people need to know, I don't need to share that, if you know what I mean
AS: yeah
FS8: for me, I don't want people saying to me how are you, what have you, I mean, my daughter, has got Crohns disease, which is a, she has had a lot of illness with that, and the thing she wishes that no so many people knew because the first thing people say, before they say anything else, is how are you and what have you got?. So she says she is just defined by her illness and not as a person and that is how I feel, its just my way, I feel as though, it is not that I feel ashamed of the fact that I have got it.
AS: in terms of the exercise content and impact? Did that have much value for you?
FS6: that would have definitely had value at the beginning, at diagnosis,

		FS35: I haven't really met others before, I had gone to a talk about an exercise class but there wasn't an opportunity to really get to know others there and I kind of found that most of the people around here were 20-30 years older than me. Whereas, FirstSteps was much more of an age related group and far better.<br>FS32: a lot of the information that was umm, given out, was stuff that you don't know as a patient, as a newly diagnosed patient. So it has benefit to form your expectations of what sort of, lies ahead if you like.<br><br>FS38: I know a number of people who have Parkinson's. umm, and there attitudes vary considerably from perhaps wanting to get involved at a local level to actually not wanting to be reminded as you just said. About how that condition might progress, in terms of the first steps program we are talking about people who have been recently diagnosed so anyone on that course may have had good or bad experiences with people with Parkinsons who are perhaps several years into that situation. But meeting people on the course who were recently diagnosed didn't expose you to that concern.
